# Combining deep sequencing and conventional molecular approaches reveals broad diversity and distribution of fleas and *Bartonella* in rodents and shrews from Arctic and Subarctic ecosystems

**DOI:** 10.1186/s13071-022-05446-w

**Published:** 2022-10-13

**Authors:** Kayla J. Buhler, Champika Fernando, Janet E. Hill, Terry Galloway, Suzanne Carriere, Heather Fenton, Dominique Fauteux, Emily J. Jenkins

**Affiliations:** 1 Department of Veterinary Microbiology, Western College of Veterinary Medicine, University of Saskatchewan, 52 Campus Drive, Saskatoon, SK S7N 5B4 Canada; 2 Department of Entomology, Faculty of Agricultural and Food Sciences, University of Manitoba, 12 Dafoe Road, Winnipeg, MB R3T 2N2 Canada; 3 Department of Environment and Natural Resources, 5Th Floor Scotiabank Centre, Government of The Northwest Territories, PO Box 1320, Yellowknife, Northwest Territories X1A 2P9 Canada; 4 Present Address: Ross University School of Veterinary Medicine, Basseterre, Saint Kitts and Nevis; 5 Centre for Arctic Knowledge and Exploration, Canadian Museum of Nature, 1740, Chemin Pink, Gatineau, QC J9J 3N7 Canada

**Keywords:** Zoonoses, *Bartonella*, Vector-borne disease, Fleas, Rodents, Arctic, Subarctic, Canada

## Abstract

**Background:**

*Bartonella* are intracellular bacteria that are transmitted via animal scratches, bites and hematophagous arthropods. Rodents and their associated fleas play a key role in the maintenance of *Bartonella* worldwide, with > 22 species identified in rodent hosts. No studies have addressed the occurrence and diversity of *Bartonella* species and vectors for small mammals in Arctic and Subarctic ecosystems, which are increasingly impacted by invasive species and climate change.

**Methods:**

In this study, we characterized the diversity of rodent fleas using conventional PCR targeting the mitochondrial cytochrome c oxidase II gene (COII) and *Bartonella* species in rodents and shrews (*n* = 505) from northern Canada using conventional PCR targeting the ITS (intergenic transcribed spacer) region and *gltA* (citrate synthase) gene. Metagenomic sequencing of a portion of the gltA gene was completed on a subset of 42 rodents and four rodent flea pools.

**Results:**

Year, total summer precipitation the year prior to sampling, average minimum spring temperature and small mammal species were significant factors in predicting *Bartonella* positivity. Occurrence based on the ITS region was more than double that of the *gltA* gene and was 34% (*n* = 349) in northern red-backed voles, 35% (*n* = 20) in meadow voles, 37% (*n* = 68) in deer mice and 31% (*n* = 59) in shrews. Six species of *Bartonella* were identified with the ITS region, including *B. grahamii, B. elizabethae*, *B. washoensis*, *Candidatus* B. rudakovii, *B. doshiae*, *B. vinsonii* subsp. *berkhoffii* and subsp. *arupensis*. In addition, 47% (*n* = 49/105) of ITS amplicons had < 97% identity to sequences in GenBank, possibly due to a limited reference library or previously unreported species. An additional *Bartonella* species (*B. heixiaziensis*) was detected during metagenomic sequencing of the *gltA* gene in 6/11 rodents that had ITS sequences with < 97% identity in GenBank, highlighting that a limited reference library for the ITS marker likely accounted for low sequence similarity in our specimens. In addition, one flea pool from a northern red-backed vole contained multiple species (*B. grahamii* and *B. heixiaziensis*).

**Conclusion:**

Our study calls attention to the usefulness of a combined approach to determine the occurrence and diversity of *Bartonella* communities in hosts and vectors.

**Graphical Abstract:**

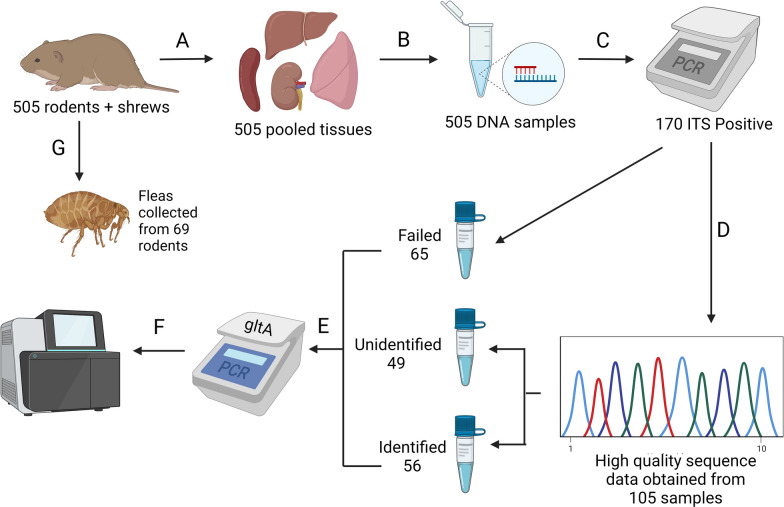

## Background

The genus *Bartonella* consists of a diverse group of emerging zoonotic bacteria that infect a range of hosts, including mammals, reptiles and birds [1–3]. These intracellular pathogens occupy endothelial cells and erythrocytes, leading to immune evasion and chronic asymptomatic bacteremia [4]. *Bartonella* infections frequently relapse following antibiotic treatment, in part due to the niches occupied outside of the blood. Several species of *Bartonella* are known to cause disease in humans and other mammals, resulting in endocarditis [5, 6], myocarditis [7, 8], splenomegaly [9] and neurological disease [10]. However, most experience mild symptoms including fever and headache [11]. The development of molecular diagnostic methods has resulted in a steady increase in the number of documented cases of bartonellosis and has drastically expanded the group of known reservoirs, vectors and *Bartonella* species [12].

Rodents are arguably the most important reservoirs for *Bartonella*, with > 98 species documented with infections [13]. There are at least 22 species of rodent-associated *Bartonella* [14], and several have been implicated in human infections, including *B. elizabethae*, *B. tribocorum*, *B. grahamii*, *B. rochalimae*, *B. vinsonii* and *B. washoensis* [13, 14]. Rodents can be co-infected with multiple species, suggesting that recombination events account for some of the diversity of rodent-associated *Bartonella* [15, 16]. Transmission occurs via blood-feeding arthropods or through the inoculation of bacteria during animal bites and scratches [17, 18]. Among the ectoparasites found on rodents, fleas are crucial vectors for *Bartonella* transmission. Under experimental conditions, rodent fleas (*Xenopsylla ramesis*) have been shown to acquire and transmit *Bartonella* spp., which suggests that rodent fleas may be competent vectors in the wild [19]. The microbiome of *Bartonella*-positive rodent fleas is also dominated by *Bartonella* lineages, suggesting that they may out-compete other community members [20]. *Bartonella* DNA has been detected in other rodent ectoparasites, including lice, ticks and mites [21, 22]; however, their vector competency is poorly understood.

Many complications can arise when trying to characterize the diversity of *Bartonella* within hosts and vectors. Co-infections are not uncommon, concentration of DNA for each *Bartonella* species can vary among tissues, recombination events can be observed in a single gene, and annealing affinity can cause primer sets to exhibit amplification bias towards species [12, 14, 23]. The citrate synthase gene (*gltA*) is the most frequently used marker for *Bartonella* spp. identification and has the largest reference library and best discriminatory power for *Bartonella* identification [12, 24]. However, some primers for the *gltA* gene can exhibit cross reactivity to host DNA (such as *Rattus* and *Mus*) and other bacterial species, reducing its analytical specificity [12, 25]. Alternatively, the intergenic transcribed spacer (ITS) region has the highest detection frequency for *Bartonella* DNA in blood and tissues [12]. There are limitations for this target, since primers may lack specificity and the locus is prone to hypervariability, containing insertions and deletions that complicate alignment and phylogenetic analysis [12, 26, 27]. In this study, we compare both targets to investigate the detection and discriminatory power for *Bartonella* spp. identification. As both conventional PCR approaches typically detect the most abundant *Bartonella* species present in samples, we also used a metagenomic approach with the *gltA* gene to identify all *Bartonella* sequence diversity within rodent hosts and vectors [16].

Despite the expansive published literature on rodent-associated *Bartonella*, no studies to our knowledge have investigated the occurrence and diversity of these pathogens in small mammals from Arctic and Subarctic ecosystems. Recently, *B. vinsonii* and *B. henselae* were reported in fleas from goose nests and Arctic foxes on the mainland of Nunavut, revealing new hosts, potential vectors and a complex web of transmission involving migratory geese and associated ectoparasites in a tundra ecosystem [28]. Thus, we determined the occurrence and diversity of endemic *Bartonella* spp. in small mammals collected from the Northwest Territories (NT) and Nunavut (NU) (Canada) and molecularly and morphologically identified the range of potential rodent flea vectors in northern ecosystems.

## Methods

### Sample collection

**Fig. 1:**
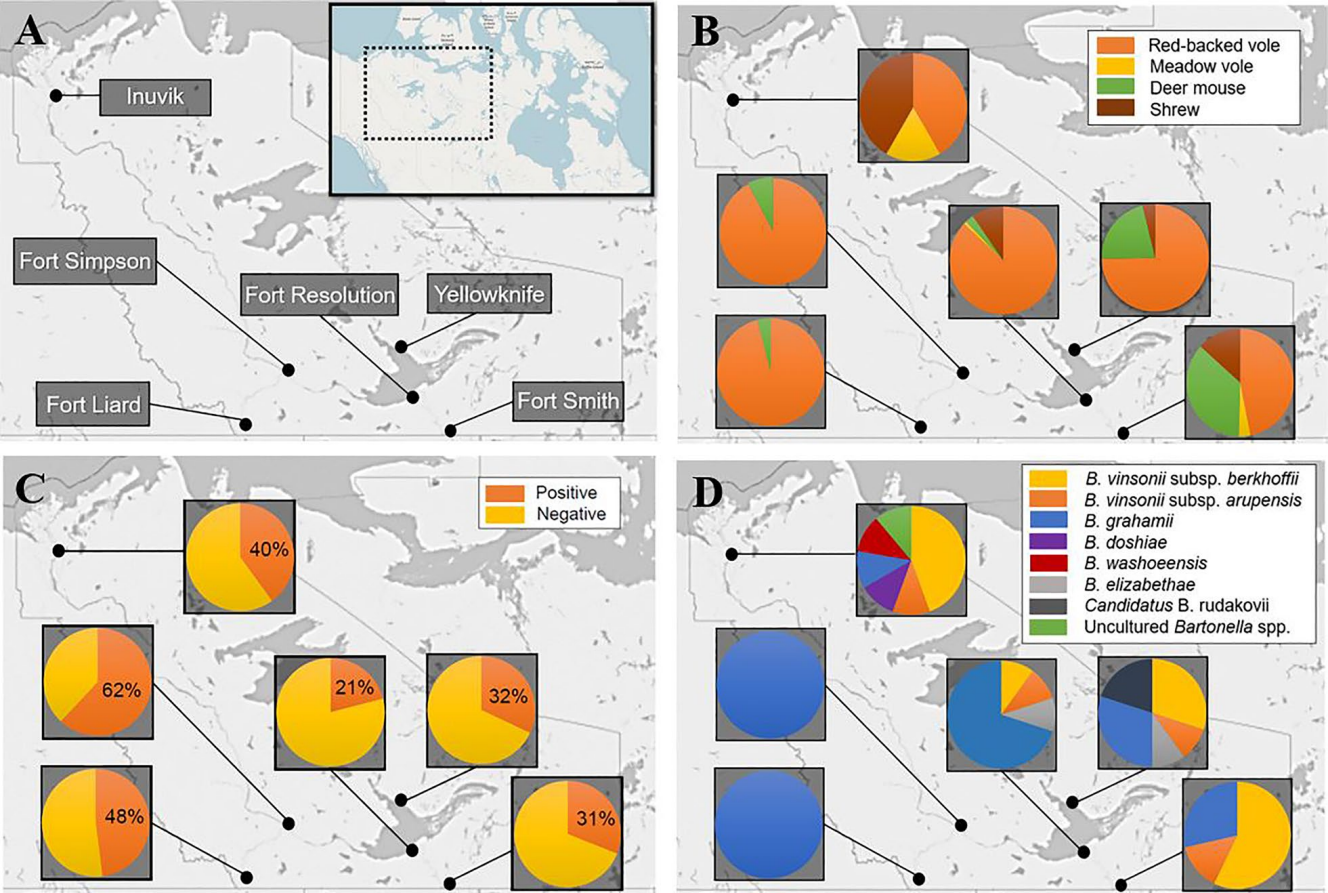
**A** Location of traplines in the Northwest Territories, Canada. **B** Composition of small mammal communities collected from each location. **C** Occurrence of *Bartonella* infections in small mammals from each location based on positive ITS PCR results. **D** Species of *Bartonella* detected in each location based on ITS sequences

**Fig. 2:**
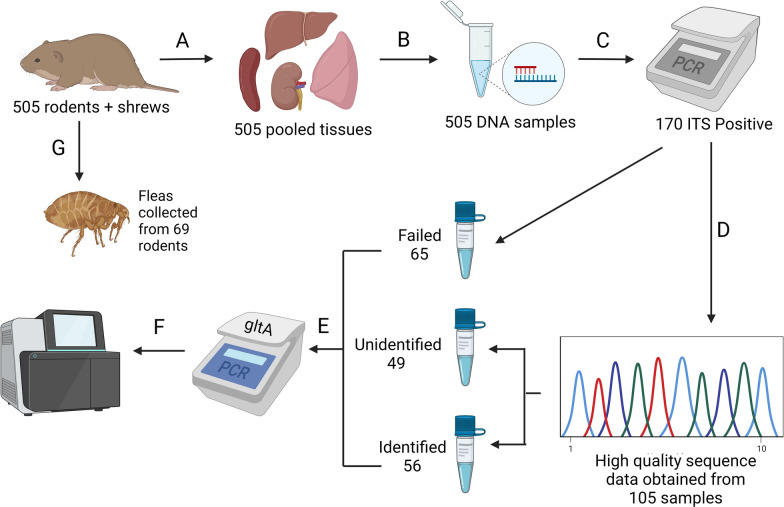
Flowchart of study methods. **A** Small mammals were necropsied, and liver, lung, spleen and kidney were collected. **B** Approximately 10 mg of each tissue was pooled, and DNA was extracted. **C** Samples were tested via a conventional PCR targeting ITS and *gltA* markers. **D** *Bartonella* species were identified via ITS amplicons and Sanger sequencing. **E** A subset of samples [including those that failed during Sanger sequencing, those that were unidentified (< 97% identity) and those that were identified (≥ 97% identity)] were selected for the modified *gltA* PCR. **F** Samples that successfully amplified underwent deep sequencing via MiSeq, and amplicon sequence variants were identified. **G** Fleas were removed from small mammals and morphologically and molecularly identified. Fleas included in the modified *gltA* PCR originated from northern red-backed voles collected from Nunavut. Created with BioRender.com

Snap-trapped rodents were collected during the summers of 2017, 2018 and 2019 using line transects placed near the communities of Yellowknife, Fort Liard, Fort Simpson, Fort Smith, Fort Resolution and Inuvik (NT, Canada) (Fig. 1). Similarly, line transects were placed near Cambridge Bay (NU, Canada) and Salluit (QC, Canada). All rodents were frozen at − 20 °C and submitted to the Zoonotic Parasite Research Unit at the Western College of Veterinary Medicine (Saskatoon, Saskatchewan, Canada). During necropsy, each rodent was morphologically identified as a northern collared lemming (*Dicrostonyx groenlandicus*), Ungava collared lemming (*Dicrostonyx hudsonius*), northern red-backed vole (*Myodes rutilus*), meadow vole (*Microtus pennsylvanicus*), deer mouse (*Peromyscus maniculatus*) or shrew. Shrews were not identified to species. Thawed carcasses were sexed and weighed and biometric measurements were collected, including length of the right hind foot, body length without the tail and body length with the tail. Any fleas found on the carcasses were pooled for each animal in 1.5-ml Eppendorf tubes containing 1 ml of 100% ethanol. The liver, lung, spleen and kidney of each animal were collected and placed in a single 1.5-ml Eppendorf tube. Organs were stored at − 20 °C until DNA extraction. No fleas were found on northern collared lemmings during our snap-trapping efforts (sample size was small); however, fleas were collected from four Ungava collared lemmings snap-trapped in Salluit. Organs from Ungava collared lemmings were not included in the study, as specimens were used for museum preparations. A flowchart outlining the methods used on small mammals is provided in Fig. 2.

### Flea identification

All fleas were identified using morphological features as per Holland [29]. For molecular identification, fleas of the same species from the same rodent were pooled together. Genomic DNA was extracted from these pools using the DNeasy Blood & Tissue Kit (Qiagen Inc; Hilden, Germany). Conventional PCR targeting ~ 615 bp of the mitochondrial cytochrome c oxidase II gene (COII) was conducted using the primers COII-2a (5’ATA GAK CWT CYC CHT TAA TAG AAC A 3’) and COII-9b (5’ GTA CTT GCT TTC AGT CAT CTW ATG 3’) [30]. A 25-µl reaction mixture was used containing 10× PCR buffer (200 mM Tris–HCl pH 8.4, 500 mM KCl; Invitrogen), 2.5 µl 50 mM MgCl_2_, 0.5 µl dNTPs (Invitrogen), 0.25 µl each primer, 0.1 µl Taq DNA Polymerase (Invitrogen) and 5 µl of genomic DNA. PCRs were conducted using the following conditions: 96 °C for 5 min followed by 35 cycles of 94° for 30 s, 55 °C for 30 s, 72 °C for 30 s and a final extension of 72 °C for 5 min. Results were visualized by agarose gel electrophoresis, and amplicons produced were then purified using the QIAquick PCR Purification Kit (Qiagen Inc.) and sequenced (Sanger sequencing; Macrogen; Seoul, South Korea).

### Identifying *Bartonella* DNA in small mammal tissues

DNA was extracted from pooled organs (liver, lung, spleen and kidney) from each individual animals using the DNeasy Blood & Tissue Kit (Qiagen Inc.). Each pooled sample was tested via conventional PCRs targeting the 16S-23S rRNA ITS region and the *gltA* gene. Positive amplification controls were gBlocks™ gene fragments (Integrated DNA Technologies; Coralville, IA, USA) designed from the complete genome of *Bartonella quintana* (BX897700.1). The primers used to amplify ~ 767 bp of the *gltA* gene were CS443f (5’ GCT ATG TCT GCA TTC TAT CA 3’) and CS1210r (5’ GAT CYT CAA TCA TTT CTT TCC A 3’) [31]. The *gltA* PCR was conducted using the following conditions: 94 °C for 2 min followed by 45 cycles of 94 °C for 30 s, 48 °C for 1 min, 72 °C for 1 min and a final extension of 72 °C for 7 min. The primers used to amplify 450–720 bp of the ITS region (size is species dependent) were 325 s (5′ CTT CAG ATG ATG ATC CCA AGC CTT CTG GCG 3′) and 1100as (5′ GAA CCG ACG ACC CCC TGC TTG CAA AGC A 3′) [32]. The ITS PCR was conducted using the following conditions: 94 °C for 5 min followed by 35 cycles of 94 °C for 1 min, 66 °C for 1 min, 72 °C for 1 min and a final extension of 72 °C for 10 min. For both PCRs, a 25-µl reaction mixture was used containing 2.5 µl 10× PCR buffer (200 mM Tris–HCl, pH 8.4, 500 mM KCl; Invitrogen), 1.25 µl 50 mM MgCl_2_, 1 µl 2 mM dNTPs (Invitrogen), 1 µl each primer (10 µM), 0.1 µl Taq DNA Polymerase (Invitrogen) and 2.5 µl genomic DNA. ITS amplicons obtained from PCR-positive samples were purified using the QIAquick PCR Purification Kit (Qiagen Inc.) and sequenced (Macrogen). No sequencing of *gltA* amplicons was performed. For ITS sequences to be identified as a species of *Bartonella*, there had to be ≥ 97% identity to sequences reported in GenBank [33].

### Metagenomic sequencing of *gltA* amplicons

A subset of 60 rodents and shrews was selected for deep sequencing of *gltA* amplicons (Fig. 2) based on whether they successfully amplified via conventional *gltA* PCR. Animals were included that were ITS PCR positive, but where Sanger sequencing of the PCR product failed, those that generated ITS sequences with < 97% identity to sequences available in GenBank and those that yielded ITS sequences that were ≥ 97% to *Bartonella* species (providing a comparison of those that had been clearly identified with the ITS region and those that were not). Four flea pools (*Amalaraeus dissimilis*) collected from northern red-backed voles in Nunavut were also selected for deep sequencing as they had previously been tested with the ITS and *gltA* PCRs. Amplification of a 487-bp fragment from the *gltA* gene was performed with primers described in Norman et al. [34] modified with the addition of Illumina adaptors: *gltA*-MiseqF (5’ TCG TCG GCA GCG TCA GAT GTG TAT AAG AGA CAG GGG ACC AGC TCA TGG TGG 3’) and *gltA*-MiseqR (5’ GTC TCG TGG GCT CGG AGA TGT GTA TAA GAG ACA GAA TGC AAA AAG AAC AGT AAA CA 3’). A 487-bp *gltA* amplicon was generated with 2 μl genomic DNA in 50-µl reactions containing 2.5 U Platinum *Taq* DNA polymerase (Invitrogen,), 2.5 mM MgCl_2,_ 50 mM KCl, 10 mM Tris/HCl pH 8.3, 250 μM each of dNTPs and 10 pmol each of primers *gltA* F and *gltA* R. Reactions were incubated at 95 °C for 3 min followed by 40 cycles of (10 s at 94 °C, 10 s at 57 °C and 30 s at 72 °C) and a final extension of 5 min at 72 °C. Amplicons were purified using 40 μl NucleoMag beads (TaKara Bio USA), and index PCR was performed according to Illumina 16S metagenomic protocol Part # 15,044,223 Rev. B with a slight modification in index PCR clean-up step by using a magnetic bead volume to 40 μl for each sample. Libraries were quantified using the Qubit ds BR kit (Invitrogen), normalized to 4 nM, pooled and loaded as an 8-pmol library with 25% phiX (Illumina) using a Miseq V2 500 cycle kit (Illumina) in a 2 × 250 sequencing run.

Amplification primer sequences were removed from demultiplexed FASTQ data using cutadapt [35], and reads were subsequently trimmed for quality with Trimmomatic [36] using a quality score of 30 and a minimum length of 100. Paired reads were merged, denoised and dereplicated using DADA2 [37] within QIIME2 (version qiime2-2019.10) using a truncation length of 200 to allow assembly of complete *gltA* amplicon sequences (~ 338 bp without primer sequences). The resulting amplicon sequence variants (ASV) and .biom files from DADA2 were exported for further analysis. The .biom files were converted to feature tables containing read counts for each unique variant detected in each sample. Within a sample, the number of reads attributed to each sequence variant was converted into a proportion of the total reads, and a specific sequence variant was only considered to be present if it accounted for ≥ 1% of the reads within a sample. Species affiliation of the ASV sequences was initially determined based on alignment of ASV to the NCBI GenBank non-redundant nucleotide database and further resolved by phylogenetic analysis of ASV sequences and *gltA* sequences selected from GenBank. Multiple sequence alignments were conducted with Muscle, and bootstrapped trees were generated using the neighbor-joining method in Mega (version 10.1.8).

### Statistical analyses

Occurrence and 95% confidence intervals (CI) were calculated using EpiTools epidemiological calculators [38]. Possible associations between predictor variables (species, sex, biometric measurements, presence/absence of fleas, weight, sampling region, year) and the outcome variable (*Bartonella* positivity) were evaluated using multiple linear regression. Similarly, associations between *Bartonella* positivity and climatic factors for both the concurrent year and the year prior to sampling were assessed via multiple linear regression (year itself was not included as a variable in these models). Climate factors included annual and seasonal values [spring (January–May); summer (June–August)] for average maximum and minimum temperature (°C) and total precipitation (mm). Data were collected from Yellowknife A Weather Station (62°27′47.000" N, 114°26′25.000" W) and obtained from the Government of Canada (climate.weather.gc.ca), as climate data from all locations in the study were not available. Models for three outcome variables were tested, including *Bartonella* positivity with (i) ITS region, (ii) *gltA* gene and (iii) positive result with both target sequences. Analyses were conducted in SPSS (version 28; IMB Corp. 2021).

## Results

### Small mammal collection

A total of 446 rodents and 59 shrews was collected, including 349 northern red-backed voles, 20 meadow voles, 68 deer mice and 9 northern collared lemmings. Most of the small mammals originated from the NT, including 82 from Yellowknife, 113 from Fort Smith, 99 from Fort Liard, 115 from Fort Resolution, 13 from Fort Simpson and 72 from Inuvik (Fig. 1). Small mammals were collected over 3 years in the NT, including the summers of 2017 (*n* = 45), 2018 (*n* = 273) and 2019 (*n* = 187). Northern collared lemmings were collected from Cambridge Bay, NU (*n* = 9). Fleas were collected from the carcasses of 69 rodents from the NT (16%; CI_95_ 12–19), including 59 northern red-backed voles (17%), 4 meadow voles (20%) and 6 deer mice (9%). Fleas from four Ungava collared lemmings were collected in Salluit (Quebec).

### Flea Identification Targeting the COII Gene

Fleas from three families were identified, including Leptopsyllidae, Ceratophyllidae and Hystrichopsyllidae. The fleas collected from small mammals are listed in Table 1. A subset of fleas was mounted on slides and kept as references [J.B. Wallis/R.E. Roughley Museum of Entomology (WRME), Department of Entomology, University of Manitoba]. Molecular identification with the COII gene verified the family and genus of most fleas collected from rodents (Table 1); however, reference data for COII sequences were limited in GenBank and species were most closely matched to species within the same genus that have ranges located further south. Following molecular identification, prepared slides were re-examined and the morphological identities of flea species verified.

**Table 1:** Morphological and molecular identification of fleas collected from northern rodents

Rodent species	Morphological Identification (number of individuals)	Molecular Identification (COII gene)	% Identity (GenBank accession)	GenBank Accession for Northern Fleas
Deer Mouse	*Aetheca thambus* (7)	*Aetheca wagneri*	98–99 (EU335975)	ON221320
	*Orchopeas leucopus* (1)	Unidentified	NA	NA
	Ceratophyllid spp. (1)	Unidentified	NA	NA
Meadow vole	Ceratophyllid spp. (1)	Unidentified	NA	NA
Red -backed vole	*Catallagia dacenkoi* (14)	*Catallagia decipiens*	83–87 (EU335987)	ON221321
	*Catallagia* sp. (3)	*Catallagia decipiens*	86 (EU335987)	ON221316
	*Peromyscopsylla selenis* (19)	*Peromyscopsylla selenis*	96–99 (KM890882)	ON221315
	*Amalaraeus dissimilis* (7)	Unidentified	NA	NA
	*Amalaraeus* sp. (5)	*Amalaraeus penicilliger penicilliger*	93–95 (KM890864)	ON221317
	*Megabothris quirini* (16)	*Megabothris groenlandicus*	96–99 (KM890796)	ON221314
	*Epitedia wenmanni* (4)	Unidentified	NA	NA
	*Rhadinopsylla linta* (2)	Unidentified	NA	NA
	Ceratophyllid sp. (3)	*Megabothris groenlandicus* *Amalaraeus penicilliger penicilliger*	99 (KM890796) 96 (KM890864)	ON221319 ON221318
	*Amphipsylla sibirica pollionis* (3)	Unidentified	NA	NA
	*Megabothris asio megacolpus* (1)	Unidentified	NA	NA
Ungava collared lemming	*Megabothris quirini* (6)	*Megabothris groenlandicus*	100 (KM890796)	ON221322

### *Bartonella* Detection by PCR Targeting *gltA* and ITS

The occurrence of *Bartonella* (ITS PCR positives) in small mammals from the NT was 34% (*n* = 170; CI_95_ 30–38). Occurrence based on *gltA* PCR (positives) was 16% (*n* = 79; CI_95_ 13–19), and only 12% of small mammals were positive on both PCRs (*n* = 60; 95% CI = 9–15). Results for *Bartonella* genetic markers for each rodent species are compared in Table 2. Unlike the ITS PCR, conventional PCR targeting the *gltA* gene did not produce any positives for deer mice or meadow voles. Occurrence in animals (ITS positives) was 22% in 2017 (*n* = 10/45; 95% CI = 13–36); 26% in 2018 (n = 72/273; 95% CI = 22–32) and 47% in 2019 (*n* = 88/187; 95% CI = 40–54).

**Table 2:** Comparison of genetic markers (ITS and gltA) used to detect *Bartonella* species in Arctic and subarctic rodents and shrews in NT, Canada, via conventional PCR

Year	Species (#)	Positives with ITS region (% [95% CI])	Positives with gltA gene (% [95% CI])	Positives with both (% [95% CI])
2017	Northern Red-backed vole (18) Deer Mouse (9) Meadow Vole (3) Shrews (15)	3 (17) 2 (22) 0 (0) 5 (15)	2 (11) 0 (0) 0 (0) 1 (7)	2 (11) 0 (0) 0 (0) 1 (7)
2017 Small mammal total	45	10 (22 [13–36])	3 (7 [2–18])	3 (7 [2–18])
2018	Northern red-backed vole (168) Deer Mouse (41) Meadow Vole (12) Collared Lemming (8) Shrews (44)	40 (24) 15 (37) 4 (33) 0 (0) 13 (30)	11 (7) 0 (0) 0 (0) 0 (0) 3 (7)	9 (5) 0 (0) 0 (0) 0 (0) 3 (7)
2018 Small mammal total	273	72 (26 [22–32])	14 (5 [3–8])	12 (4 [3–8])
2019	Northern red-backed vole (163) Deer Mouse (18) Meadow Vole (5) Collared Lemming (1)	77 (47) 8 (44) 3 (60) 0 (0)	62 (38) 0 (0) 0 (0) 0 (0)	45 (28) 0 (0) 0 (0) 0 (0)
2019 Small mammal total	187	88 (47 [40–54])	62 (33 [27–40])	45 (24 [19–31])
Project total	505	170 (34 [30–38])	79 (16 [13–19])	60 (12 [9–15])

Of the 105 ITS PCR products that yielded high-quality sequence data (ranging from 230 to 606 bp), only 56 (53%) provided a species identification (≥ 97% identity with *Bartonella* sequences in GenBank, Fig. 2). The most common species of *Bartonella* identified in both northern red-backed and meadow voles was *B. grahamii* (Table 3). In deer mice, the most common species detected were *B. vinsonii* subsp. *arupensis* and *B. grahamii*. In shrews, the most common species detected was *B. vinsonii* subsp. *berkhoffii.* The occurrence of *Bartonella* (ITS positives) in small mammals for each location sampled in the NT was 48% for Fort Liard (*n* = 47/99; 95% CI = 38–57), 21% for Fort Resolution (*n* = 24/115; 14–29), 62% for Fort Simpson (*n* = 8/13; 36–82), 31% for Fort Smith (*n* = 35/113; 23–40), 40% for Inuvik (*n* = 29/72; 30–52) and 32% for Yellowknife (*n* = 26/82; 23–42) (Fig. 1).

**Table 3:** *Bartonella* species detected with the ITS PCR in rodents and shrews from the Northwest Territories

Small mammal species^1^	Number (%)	Species identification^2^
Red backed voles (*n* = 69)	1 (1.5) 28 (40.6) 3 (4.3) 1 (1.5) 1 (1.5) 2 (2.9) 2 (2.9) 31 (44.9)	*B. doshiae* *B. grahamii* *B. vinsonii* subsp. *berkhoffii* Uncultured *Bartonella* spp. *B. washoeensis* *B. elizabethae* *Candidatus* B. rudakovii Unidentified
Deer mice (*n* = 18)	3 (16.7) 1 (5.6) 3 (16.7) 11 (61)	*B. vinsonii* subsp. *arupensis* *B. vinsonii* subsp. *berkhoffii* *B. grahamii* Unidentified
Meadow voles (*n* = 5)	2 (40) 3 (60)	*B. grahamii* Unidentified
Shrews (*n* = 13)	8 (61.5) 1 (7.7) 4 (30.8)	*B. vinsonii* subsp. *berkhoffii* *B. vinsonii* subsp. *arupensis* Unidentified

^1^Animals for which ITS PCR was positive and high-quality sequence data were obtained are included

^2^Species identified based on ITS sequences with ≥ 97% identity. Undetermined species are those that had < 97% identity with sequences available in GenBank

### Factors associated with occurrence of *Bartonella*

Year (*β* = 0.2, 95% CI = 0.1–0.3) was significantly associated with *Bartonella* positivity in small mammals across all three models, with the highest exposure in 2019. No additional biological factors were associated with small mammal exposure in the model that defined *Bartonella* positivity as a PCR positive result via the ITS region (*R*^2^ = 0.06, df = 1, *P* < 0.001). Rodent species (*β* = 0.1, 95% CI = 0.03–0.1) became statistically significant alongside year (*β* = 0.2, 95% CI = 0.1–0.3) in the model that defined *Bartonella* positivity with the *gltA* gene (*R*^2^ = 0.1, df = 2, *P* = 0.001). Similarly, when *Bartonella* positivity was defined as a positive result on PCR assays for both the ITS and *gltA* markers, rodent species (*β* = 0.1, 95% CI = 0.02–0.09) and year (*β* = 0.1, 95% CI = 0.09–0.2) were statistically significant (*R*^2^ = 0.09, df = 2, *P* = 0.005). Across all three models, total summer precipitation for the year prior to sampling and average minimum spring temperature during the year of sampling were statistically significant [ITS (R^2^ = 0.05, df = 2, *P* < 0.001), *gltA* (*R*^2^ = 0.1, df = 2, *P* < 0.001), PCR positive for both (*R*^2^ = 0.08, df = 2, *P* < 0.001)], with colder minimum spring temperatures and higher precipitation during the year prior to sampling linked to more PCR-positive small mammals.

### Metagenomic sequencing of the *gltA* region

Of the 60 small mammal samples and 4 flea pools selected for metagenomic gltA amplicon sequencing, 44 of the small mammals (73%) and all flea samples successfully amplified on the modified gltA PCR. Our two no-template controls yielded 0 and 67 reads. An average of 12,272 raw reads per sample were obtained (median 11,696; range 707 to 27,116). Following quality filtering, two northern red-backed voles were removed from further analysis because of extremely low sequence read counts (< 50 reads) leaving 46 samples (42 rodents, 4 flea pools). Twenty-two unique amplicon sequence variant (ASV) sequences were identified. Four ASV sequences were removed from further analysis since they were determined not to be gltA sequences based on alignment to reference data. The average total ASV count per sample was 5662 (median 5642, range 337 to 13,974).

**Fig. 3:**
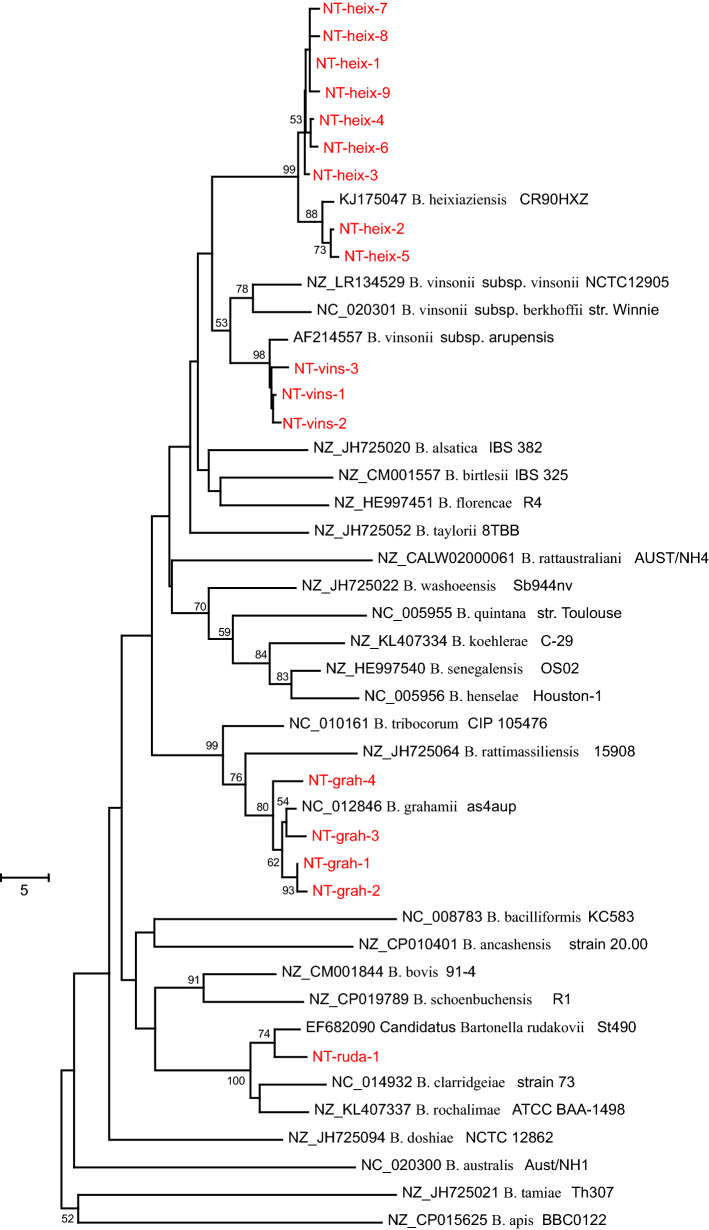
Phylogenetic relationships of *gltA* sequence variants with previously published reference sequences. Study sequences amplified from small mammals and flea pools are shown in red. Accession numbers for reference sequences are indicated. The tree was constructed from a 309-bp alignment using the neighbor-joining method. The tree is drawn to scale, with branch lengths indicating differences per sequence according to the scale bar. The percentages of replicate trees in which the associated taxa clustered together in the bootstrap test (500 replicates) are shown next to the branches

Seventeen unique ASVs were detected that accounted for at least 1% of the counts in at least one sample. A phylogenetic analysis was performed based on a 309-bp alignment of the *gltA* ASV sequences and selected reference sequences. Four of the ASVs clustered with *B. grahamii*, nine with *B. heixiaziensis*, one with *Candidatus* B. rudakovii and three with *B. vinsonii,* all with good bootstrap support (Fig. 3). Four ASVs (NT-heix-7, NT-heix-8, NT-heix-9 and NT-grah-4) were detected only in fleas. ASV sequences resembling *B. grahamii* were most common, detected in 28 of the 42 rodents and accounting for 100% of sequence reads in each sample. There was 89% agreement for sequences that were identified as *B. grahamii* on both ITS and modified *gltA* PCRs (18 rodents via ITS PCR and 16 of the same rodents via modified *gltA* PCR) (Table 4). ASVs resembling *B. heixiaziensis* were the second most common variants, detected in 11 of the rodent samples (100% of counts in each sample). There was no agreement between sequences obtained via ITS and modified *gltA* PCRs for these rodents, likely because of the absence of ITS sequences for *B. heixiaziensis* in GenBank. There was no agreement between ASVs that resembled *B. vinsonii* and *Candidatus* B. rudakovii, despite the availability of ITS sequences in GenBank. Multiple species were not detected in rodents, and only one flea pool contained more than one species (*B. grahamii* and *B. heixiaziensis*). Of the 46 samples that underwent deep sequencing, 24 (52%) contained multiple ASVs.

**Table 4:** Comparing rodents collected in NT, Canada (2017–2019), that underwent Sanger sequencing following conventional PCR (ITS region) and metagenomic sequencing following modified gltA PCR. Only amplicon sequence variants accounting for at least 1% in a sample were included

Location	Rodent species^1^	*Bartonella* species (Sanger sequencing)	% Identity	Accession number (ITS)	*Bartonella* species (metagenomic sequencing)	% Identity	Accession number (gltA)	Amplicon sequence variants
	RBV	Unidentified	91	KX420734	*B. heixiaziensis*	98	KJ175047	NT-heix-1 NT-heix-4
Fort Resolution	RBV	*B. grahamii*	99	MH687377	*B. grahamii*	99	NC_012846	NT-grah-1
	RBV	*B. elizabethae*	100	LR134527	*B. heixiaziensis*	99	KJ175047	NT-heix-3 NT-heix-6
	RBV	*B. grahamii*	99	MH687377	*B. grahamii*	99	NC_012846	NT-grah-1
	DM	Unidentified	90	KU292577	*B. vinsonii*	99	AF214557	NT-vins-1 NT-vins-2 NT-vins-3
	RBV	*B. grahamii*	99	MH547345	*B. heixiaziensis*			NT-heix-1 NT-heix-4
Yellowknife	RBV	Failed	Failed	Failed	*B. heixiaziensis*			NT-heix-1 NT-heix-4
	RBV	*B. vinsonii*	99	AF167988	*B. heixiaziensis*	98	KJ175047	NT-heix-1 NT-heix-4
	RBV	*Candidatus* B. rudakovii	100	EF682087	*B. heixiaziensis*			NT-heix-1 NT-heix-4
	RBV	Unidentified	96	KX420719	*B. heixiaziensis*			NT-heix-1 NT-heix-4
Fort Smith	RBV	Unidentified	95	DQ683199	*Candidatus *B. rudakovii	98	EF682090	NT-ruda-1
	RBV	Unidentified	94	KX420620	*Candidatus *B. rudakovii			
Fort	RBV	Failed	Failed	Failed	*B. grahamii*	99	NC_012846	NT-grah-1
Simpson	RBV	*B. grahamii*	98	MH687377	*B. grahamii*			
	RBV	Unidentified	94	AB602558	*B. heixiaziensis*			NT-heix-2 NT-heix-5
	RBV	Unidentified	87	AB602558	*B. heixiaziensis*			NT-heix-2 NT-heix-5
Inuvik	RBV	Unidentified	88	MH477609	*B. heixiaziensis*	99	KJ175047	NT-heix-2 NT-heix-5
	RBV	Unidentified	94	AB602558	*B. heixiaziensis*	98 99 99	KJ175047	NT-heix-1 NT-heix-2 NT-heix-5
	RBV	*B. grahamii*	99	AJ269785				NT-grah-1
	RBV	*B. grahamii*	97	CP001562				NT-grah-1 NT-grah-2
	RBV	*B. grahamii*	99	MH687377				NT-grah-1
	RBV	*B. grahamii*	98	MH547345				NT-grah-1
	RBV	*B. grahamii*	99	MH547345				NT-grah-1
	RBV	*B. grahamii*	98	MH687377				NT-grah-1
	RBV	Failed	Failed	Failed				NT-grah-1
	RBV	Failed	Failed	Failed				NT-grah-1
	RBV	*B. grahamii*	99	MH687377				NT-grah-1 NT-grah-2
	RBV	Failed	Failed	Failed				NT-grah-2
	RBV	*B. grahamii*	98	MH687377				NT-grah-1 NT-grah-2
	RBV	*B. grahamii*	97	MH547345				NT-grah-1
Fort Liard	RBV	*B. grahamii*	99	MH547345	*B. grahamii*	99	NC_012846	NT-grah-1
	RBV	*B. vinsonii*	90	MK773863				NT-grah-1
	RBV	Unidentified	95	AJ269785				NT-grah-1 NT-grah-2
	RBV	*B. grahamii*	99	MH547345				NT-grah-3
	RBV	Failed	Failed	Failed				NT-grah-2
	RBV	Failed	Failed	Failed				NT-grah-1 NT-grah-2
	RBV	Failed	Failed	Failed				NT-grah-1
	RBV	Failed	Failed	Failed				NT-grah-1
	RBV	*B. grahamii*	98	MH547345				NT-grah-1 NT-grah-2
	RBV	Failed	Failed	Failed				NT-grah-1
	RBV	*B. grahamii*	97	AJ269785				NT-grah-2
	RBV	Failed	Failed	Failed				NT-grah-1 NT-grah-2

^1^*RBV* Northern red-backed vole, *DM* deer mouse

Sequence data have been deposited to the NCBI Sequence Read Archive and are associated with BioProject PRJNA813524.

## Discussion

This study supports the diversity of fleas on northern small mammals and identifies a complex community of rodent-associated *Bartonella* present in northern Canada, demonstrating the utility of conventional and metagenomic approaches. Ten distinct flea species from rodents were identified morphologically. Molecular identification with the COII gene verified the genus of most flea species (*n* = 9/10) and highlights the need for a larger reference library for northern vectors (Table 1). Our morphological identifications are consistent with the known geographic range and the time of year that samples were collected [29]. All flea species that were identified infesting small mammals from the NT and NU have been previously recorded on small mammals from Alaska and Yukon [39]. The list of flea species likely contains competent vectors for *Bartonella*, as rodent fleas can transmit the bacteria under experimental conditions [19, 31, 40]. All rodent flea pools (*Amalaraeus dissimilis*) that underwent deep sequencing contained *Bartonella* DNA, though this does not provide definitive proof of vector competence. As seen with *B. henselae* and cat scratch disease, a source of flea-borne transmission involves the indirect inoculation of infected flea feces, which can remain viable for up to 72 h, into cuts or abrasions on the skin [41–44]. Given the diversity of *Bartonella* spp. identified in small mammals and fleas during this study, similar passive mechanisms of transmission may be involved. *Bartonella* bacteria are dominant members of bacterial communities in several rodent fleas and mixed infections occur in the rat flea, *Nosopsyllus fasciatus* [16, 20]. This supports the ‘spillover phenomenon,’ where *Bartonella* transmission is driven by interspecies interactions and exchange of fleas with low host specificity [13]. Though our study did not test individual fleas for mixed infections, our results (deep sequencing) indicate that one flea pool contained two species of *Bartonella*, revealing that conventional molecular methods underestimate *Bartonella* diversity in pooled vector samples.

Conventional PCR targeting the ITS region identified six species of *Bartonella* in rodents and shrews from the NT (Table 3). Rodents are known reservoirs for many of these species and often develop asymptomatic and persistent infections [19]. All *Bartonella* spp. detected via ITS PCR are zoonotic and have been associated with human cases of disease, except for *Candidatus* Bartonella rudakovii [11]. In addition, several have been shown to infect domestic dogs, including *B. vinsonii* subsp. *berkhoffii*, *B. elizabethae* and *B. washoensis* [45, 46]*.* Cases of bartonellosis (B. *vinsonii* subsp. *berkhoffii*, *B. henselae* and *B. rochalimae*) have also been identified in Arctic foxes from the neighboring territory of Nunavut, Canada [28, 47]. To our knowledge, there have been no published reports of human bartonellosis in the NT, as it is not a reportable disease.

The occurrence of *Bartonella* (ITS PCR positives) in small mammals from northern Canada was 34% (Table 2), which is lower than numbers previously reported in wild rodents in the southern Canadian province of Saskatchewan (57%) [48]. *Bartonella grahamii* was the most common species detected in red-backed voles by our study and Jardine et al. [48]. In our study, small mammal species appeared to influence *Bartonella* spp. diversity, as *B. grahamii* was more prevalent in voles while *B. vinsonii* was more prevalent in deer mice and shrews. This may reflect differences in habitat use, interspecies interactions and variability in host specificity of ectoparasites. Our location data support this hypothesis, as there were significant differences in distribution of small mammals and *Bartonella* spp. among sampling sites in the NT (Fig. 1). Additionally, multiple ASVs for the *gltA* gene were only detected in one geographic location (for example NT-heix-3 and NT-heix-6 in Fort Resolution or NT-heix-2 and NT-heix-5 in Inuvik; Table 4), indicating that shared environments may influence *Bartonella* diversity. As the climate continues to warm in northern Canada, the species that make up these small mammal communities, along with their associated *Bartonella* spp., may change with shifts in habitat use and the movement of southern competitors further north [49, 50].

Average minimum temperature during spring (°C) and total summer precipitation during the year prior to rodent sampling (mm) were associated with *Bartonella* positivity in small mammals across all three models. Occurrence was higher during years with lower spring temperatures and years that followed summers with higher precipitation. These results must be interpreted with caution, as climate data were collected from one weather station (Yellowknife), which may not accurately represent other sampling sites. Regardless, climate change is likely to impact the survival of both ectothermic insects and small mammals [51, 52]. Rodent-associated *Bartonella* has previously been found to be density dependent [53], and climate factors known to influence rodent survival were associated with *Bartonella* positivity in our study [54]. There was greater precipitation (total mm) in Yellowknife during the summer of 2018 compared to other sampling years (> 100 mm more than 2017 and 2019), which was associated with increased *Bartonella* positivity in small mammals during 2019. Rainfall at critical times could cause flooding of rodent habitats and burrows, increasing interactions due to limited habitat as rodents search for higher ground [55, 56]. Northern red-backed voles made up the largest percentage of our specimens (69%), and previous studies have proposed that high summer precipitation leads to a greater abundance of late summer and fall berry crops (a key food source for voles) [57]. In turn, this may lead to good overwinter survival and benefit summer reproduction the following year [58, 59]. Consequently, high precipitation during the summer of 2018 may have promoted overwintering survival of northern red-backed voles (and potentially other small mammals) and caused higher population density during 2019, thus increasing the probability of transmission of ectoparasites and *Bartonella*. Lower spring temperatures may also lead to high rodent density as delayed snowmelt may provide longer protection in subnivean spaces and promote rodent survival [54, 59].

Fleas are ectothermic and sensitive to changes in temperature and precipitation [60]; however, there are few studies in which the impacts of climate on flea populations in northern ecosystems have been examined. Eggs, larvae and pupae of fleas occur off the host within rodent nests, which can reduce the effects of temperature fluctuations [61]. Presumably, this buffer effect may be even more pronounced in northern ecosystems because of the insulating layer of snow. Soil moisture within rodent burrows created by outside precipitation influences the survival of pre-adult stages of flea [60]. Increased rainfall during the summer of 2018 may have increased survival of immature stages, leading to an increase in the number of *Bartonella* positive small mammals during the summer of 2019. The presence of fleas on rodent carcasses was not significantly associated with PCR positive animals across all three models (*gltA*, ITS and both). However, snap-trapped rodents are often collected up to 24 h after death, providing a window of time for ectoparasites to abandon the host, which likely caused the prevalence and abundance of fleas in our study to be underestimated.

Rodent species were not statistically significant in the first model that defined *Bartonella* positivity as a positive ITS result. However, it was significant in the two models that defined *Bartonella* positivity as a positive *gltA* result or a positive result on both conventional PCRs. Interpreting associations between rodent species and *Bartonella* positivity is complicated in this case as primers targeting some genetic markers may amplify host DNA and sensitivity of the conventional *gltA* PCR may be lower than that of the ITS PCR (Table 2) [12].

Only 62% (*n* = 105) of the 170 positive small mammal samples identified on the ITS PCR were successfully sequenced and 47% (*n* = 49/105) of these sequences had < 97% identity with previously reported sequences in GenBank. While it is possible that the low sequence identities were due to known challenges with ITS sequence alignment or the identification of uncharacterized species, we hypothesized that these were likely due to the limited reference library available for ITS sequences [12, 27]. Ideally, multiple loci are used to identify new *Bartonella* species [12]. Hence, a subsample of rodents was tested with a modified *gltA* PCR and metagenomic sequencing. There was high agreement between the ITS and *gltA* targets for most rodents that were infected with *B. grahamii* (Table 4). One additional species (*B. heixiaziensis*) was detected with the metagenomic approach (Fig. 3, Table 4). This species was originally isolated from the blood of a red-backed vole (*Myodes rutilus*) on Heixiazi Island (on the border of China and Russia) [62]. To our knowledge, this is the first documentation of *B. heixiaziensis* in North America. *Bartonella heixiaziensis* was the second most common species detected with deep sequencing, and there were no ITS sequences available in GenBank. Thus, we suggest that the absence of reference sequence data for the ITS region accounts for many of the specimens with low sequence similarity following conventional PCR (Table 4). During this study, two unique sequences for the ITS region were isolated from rodents that contained *B. heixiaziensis* DNA via deep sequencing of the *gltA* gene. These sequences are available in GenBank (accession numbers ON226744 and ON226745).

## Conclusion

We found a diversity of previously undescribed rodent-associated *Bartonella* in northern Canada and a complex community of potential flea vectors present on rodents that may play a role in transmission. The majority of *Bartonella* spp. detected in this study are zoonotic and may contribute to human morbidity in the North (associated with exposure to vectors or reservoir hosts) [11]. In addition, we identified climatic factors associated with *Bartonella* positivity and addressed previously reported strengths and weaknesses of conventional PCR with genetic loci (*gltA* and ITS) commonly used for detection and identification of *Bartonella* spp. A metagenomic approach was used to detect one additional species of *Bartonella* (*B. heixiaziensis*) in northern red-backed voles that was previously unreported in North America and demonstrated that northern flea pools can contain multiple bacterial species. Future studies that aim to describe the composition of *Bartonella* communities in hosts and vectors accurately should use multiple loci to identify species and implement a metagenomic approach to detect the diversity of *Bartonella* within samples.

## Data Availability

The datasets supporting the conclusions of this article are available in the Zenodo repository doi: 10.5281/zenodo.6332993. Sequences for *B. heixiaziensis* (accession numbers ON226744 and ON226745) and rodent fleas (ON221314—ON221322) are available in GenBank.
